# Use of Plant Proteins as Microencapsulating Agents of Bioactive Compounds Extracted from Annatto Seeds (*Bixa orellana* L.)

**DOI:** 10.3390/antiox9040310

**Published:** 2020-04-13

**Authors:** Julián Quintero Quiroz, Víctor Velazquez, Ligia Luz Corrales-Garcia, Juan D. Torres, Efren Delgado, Gelmy Ciro, John Rojas

**Affiliations:** 1 Facultad de Ciencias Farmacéuticas y Alimentarias, Universidad de Antioquia, Calle 67 No. 53-108, University Campus, Medellín 050010, Colombia; ligia.corrales@udea.edu.co (L.L.C.-G.); juan.torreso@udea.edu.co (J.D.T.); gelmy.ciro@udea.edu.co (G.C.); jhon.rojas@udea.edu.co (J.R.); 2 Department of Family and Consumer Sciences, College of Agriculture, Consumer and Environmental Sciences, New Mexico State University, NMSU Gerald Thomas Hall Room, 308 P.O. Box 30003 MSC 3470, Las Cruces, NM 88003, USA; yambe@nmsu.edu (V.V.); edelgad@nmsu.edu (E.D.)

**Keywords:** bixin, ionic gelation, lentil proteins, polyphenol compounds, quinoa proteins

## Abstract

This study aimed to assess the thermal stability of the bioactive compounds from annatto seed extract, encapsulated by ionic gelation using quinoa proteins, lentil proteins, soy proteins, and sodium caseinate as carrying materials. The 10.0% aqueous dispersions of the different proteins (carriers) were prepared and mixed with the annatto seed extract. The dispersions were then extruded into a calcium chloride solution to induce the extract encapsulation. The capsules were characterized by encapsulation efficiency, particle size, infrared transmission spectroscopy, confocal microscopy, and scanning electron microscopy (SEM). The antioxidant and antimicrobial activities, the polyphenol compounds, and bixin content from the free and encapsulated extract were assessed once stored for 12 d at different temperatures (4 °C, 25 °C, and 65 °C). The results demonstrated the ability of the proteins to encapsulate the annatto extract with encapsulation efficiencies ranging from 58% to 80%, where the protein structure and amino acid content were the relevant factors to obtain high encapsulation efficiencies. The free extracts stored at 65 °C for 12 d experienced a degradation of bixin and polyphenol compounds, respectively. Conversely, the encapsulated extract had degradations from ~34.00% to ~4.05% for polyphenol compounds and ~20.0% for bixin, respectively. These proteins have a potential encapsulation capacity of annatto extract by ionic gelation.

## 1. Introduction

The extracts obtained from the seeds of annatto (*Bixa orellana* L.) have been remarkable due to their coloring capacity and antioxidant activity. Some bioactive compounds derived from annatto seeds have also shown antimicrobial and antioxidant activities of particular interest for the preparation of food products. In fact, these extracts have been used in food matrices such as dairy, meat, and baked products, mostly due to their coloring property [1,2]. The antioxidant and antimicrobial activities of the extract are attributed to the bioactive compounds found in the seeds, such as the bixin carotenoid (bixin or 6-methyl hydrogen (9*Z*)-6,6′-diapocarotene-6) and polyphenol compounds such as catechin, chlorogenic acid, chrysin, butein, hypoaletin, and xanthoangelol [3]. These compounds inhibit the production of free radicals and denature the proteins present in the cellular structure of some microorganisms to prevent their proliferation [1,4,5,6]. However, the use of these antioxidant or antimicrobial compounds in food products is still limited because of their low stability. The sensitivity of the bioactive compounds from the extract to oxygen, light, and temperature implies the inclusion of a protective agent to potentiate the beneficial effects of the extract [1].

The encapsulation process provides physical protection of the bioactive compounds from the external conditions such as high temperatures, pressure changes, moisture, oxygen, or light, increasing the stability of the bioactive compounds and enhancing their life span [7]. Spray-drying, coacervation, freeze-drying, ionic gelation, liposomes, and emulsions are the main encapsulation techniques used for the protection of bioactive compounds [7]. Ionic gelation takes advantage of the chemical structure from the coating material to encapsulate bioactive compounds in a 3D gel-like system. This technique is a promising encapsulation technique for encapsulating hydrophilic and hydrophobic compounds due to its simplicity and low cost, and because it does not require the use of organic solvents or high temperatures compared with other encapsulation techniques such as freeze-drying, coacervation, or spray-drying [7,8]. In turn, ionic gelation implies the extrusion of a dispersion containing the ionic coating material and the bioactive compounds onto a gelling solution rich in counter ions. For instance, several bioactive compounds have been encapsulated with a sodium alginate dispersion once extruded onto a calcium chloride solution. The gelation phenomenon is triggered when calcium ions react with the free carboxyl moieties present in the alginate structure by ion crosslinking, forming hydrogels, and thus trapping the bioactive substances [7,9]. The extrusion method mostly produces capsules with diameters of several mm dominantly controlled by rheological properties of the polymer solution and the extrusion system tip diameter [10,11]. To fabricate capsules by ionic gelation in a more controllable manner, techniques such as the electrospraying (electrohydrodynamic atomization) method have gained great attention, but traditional extrusion techniques continue to be used to assess the gelation capacity of different coating materials. Other materials such as low metoxilation pectin, chitin, and chitosan have been reported as carrier agents in the microencapsulation process by ionic gelation in systems for food compounds and controlled release, as they are highly biocompatible, not toxic, and mechanically strong [8].

The study of proteins as coating materials in encapsulation processes has increased significantly in recent years. In microencapsulated products, proteins function simultaneously as a coating material and a source of amino acids [12]. In addition to their nutritional contribution, proteins have various functional properties, such as the ability to stabilize colloidal systems, modify the rheological properties of the medium, retain water or oil in the system, and form gels and foams [13]. The functional properties are attributed to the structure of the proteins. In turn, this flexible and changing structure depends on the medium conditions, the presence of lipophilic and hydrophilic microdomains distributed throughout their structure (amphiphilic property), and the net protein charge [13].

Currently, the most common use of plant-derived proteins is focused on the agricultural and food fields, particularly in emerging market niches such as vegan foods. Proteins from plant sources are considered isolated macromolecules from highly renewable and abundant sources, which can reduce the spread risk of animal-borne diseases, and its uses and potentization are among the current market trends [14,15]. Therefore, several studies have dealt with the isolation of proteins from plant sources such as quinoa, peas, soybeans, rice, sunflowers, and pumpkin seeds. As a result, suitable emulsifying activity and improved stability, gel formation capacity, and encapsulating ability have been found following several techniques [16,17,18,19,20].

Various authors have studied the capacity of capsule formation using animal proteins such as sodium caseinate and protein isolates from soy, lentils, sunflower seeds, peas, and beans [16,19,20,21,22,23,24,25,26]. Those authors used plant proteins as encapsulation agents of vitamins, oils with antioxidant activity, and plant extracts, mainly by the methods of coacervation and spray-drying [16,19,20,21,22,23]. The gelling properties of proteins are established on their ability to form three-dimensional networks, and this depends upon the tertiary structure change of the proteins, either by a partial denaturation caused by thermal treatments or a change in their structure by the breaking of peptide links [27]. This structural modification allows the formation of new electrostatic and hydrophobic interactions, hydrogen bonding, and disulfide bonds between the denatured protein subunits. Glutaraldehyde is the most commonly used crosslinking agent to obtain stable microcapsules, and microcapsules with better mechanical properties employed proteins as the carrier material [28,29]. However, glutaraldehyde is a relatively toxic product, which limits its use in applications such as the food industry [19]. The presence of Ca^2+^ ions during the formation of the protein gels increases coordinated ionic interactions between free carboxyl moieties present in the protein structure (amino acids such as aspartic and glutamic acid) and Ca^2+^ ions, thereby strengthening the gel structure [27].

In vitro studies employed a mixture of soy protein and sodium alginate as the coating materials to encapsulate thyme essential oil by ion gelation [30]. Those authors showed that soy protein increased the encapsulation efficiency from 76% to 80% based on total polyphenol compounds and decreased the particle size from 1312 ± 32 µm to 1256 ± 51 µm. The changes were attributed to the exposed soy protein radical groups generating a net-like structure with Ca^2+^ ions [30].

Other studies conducted on globular proteins showed that soy and whey proteins are efficient encapsulation agents to protect and modulate the release of a wide variety of nutraceuticals and bioactive compounds [12]. Those results motivated the search for new protein sources with potential gelling capacity, as well as the ability to function as encapsulating agents of various bioactive compounds via hydrogel systems, and to evaluate the in vivo release mechanisms of digestive systems [12].

Quinoa (*Chenopodium quinoa*) is a pseudo-cereal grown mainly in the Andes of South America and contains between 12% and 20% protein. These proteins are composed of globulins and albumins with a sedimentation coefficient of 37% and 35% for the 11S and 2S subunits, respectively (weight between 8 and 39 kDa). Quinoa is rich in essential amino acids, especially lysine, which is virtually absent in some grains and wheat [31]. Lentils (*Lens culinaris*), on the other hand, are legumes having from 24% to 30% protein content depending on the genotype and growing conditions [32]. Likewise, their proteins correspond to the globulin and albumin type, with a molecular weight ranging from 14 to 66 kDa. Some studies have reported that proteins isolated from lentils have a high emulsifying capacity along with ample water and oil absorption capacities [33,34]. Plant proteins can be used as coating materials, but the ionic gelation encapsulation process has attracted only a few research studies. Therefore, the goals of this study were to (i) encapsulate the annatto seeds extract via ionic gelation, using different plant proteins as coating materials, and (ii) assess the effect of temperature on the antioxidant and antimicrobial activity of the encapsulated extract.

## 2. Materials and Methods

### 2.1. Materials

Sodium carbonate (Lot 94F-0313), sodium acetate trihydrate (Lot SLBX1773), absolute ethanol (lot SHBK8928), concentrated hydrochloric acid (SHBJ6587), 2,4,6-tri-(2-pyridyl)-1,3,5-triazine (lot BCBW9015), 2,2-diphenyl-1-picrylhydrazil (lot BCBQ6979), sodium citrate (lot BCBQ6979), bixin standard (lot BCBB0915), acetone (lot BCBQ6979), nitroblue tetrazolium (NBT) (lot BCBQ6979), nicotinamide adenine dinucleotide (NAD) (lot BCBQ6979), and calcium chloride (lot 08K0066) were obtained from Sigma-Aldrich (St. Louis, MO, USA). Ferric chloride.6H_2_O (lot 176057) and dimethyl sulfoxide (lot 183521) were purchased from Fisher Chemical (Sunnyvale, CA, USA). In addition, (±)-6-hydroxy-2,5,7,8-tetramethylchromane-2-carboxylic acid (Trolox^®^) (97%, lot A0405014) and gallic acid (A0267865) were acquired from Acros Organics (Morris Plains, NJ, USA). Nutrient broth (lot 7121869) and ethanol (K18G24K10), tris-HCL molecular biology grade (lot 0000308001)) were obtained from Becton Dickinson (Franklin Lakes, NJ, USA), Pharmco (Titusville, FL, USA), and Promega (Madison, WI, USA), respectively. Additionally, 3-(4,5-dimethylthiazol-2-yl)-2,5-diphenyl-tetrazolium bromide (MTT) (lot p31b064) and ammonium sulfate were purchased from Alfa Aesar (Haverhill, MA, USA) and Bell Chem International (Medellin, Colombia), respectively.

Soy protein (SP) isolates and sodium caseinate (SCP) used as controller were obtained from Bell Chem International S.A.S (Medellin, Colombia). Annatto seeds were donated by a production farm located in the department of Córdoba, Colombia (63 m above sea level). The seeds of quinoa and lentils were obtained from a farmer market in Medellín, Colombia. The fresh seeds were oven-dried (model IMP180, Thermo Fisher Scientific) at 37 °C for 48 h, subsequently milled and passed through a number 60 mesh (250 µm size), and stored in desiccators until use.

### 2.2. Annatto Seed Extract

The bioactive compounds were extracted from the annatto seeds with 80% ethanol at a 1:5 *w*/*v* ratio for 48 h under constant stirring [35]. The extract thus obtained was concentrated at 60 mbar and 35 °C using a rotary evaporator (R-114, BÜCHI^®^ Rotary evaporator, New Castle, DE, USA) and freeze-dried. Total polyphenol content, antioxidant activity (ferric reducing antioxidant power (FRAP) and superoxide radical scavenging activity (SRSA)), antimicrobial activity, and bixin content were determined in the freeze-dried sample as described below.

#### 2.2.1. Total Polyphenol Compounds

The polyphenol content was determined using the Folin–Ciocalteu reagent [36]. First, 100 µL of Folin–Ciocalteu reagent, 300 µL of 20% sodium carbonate solution, 1.58 mL of deionized water (DI water), and 20 µL of the sample (~0.1 g of freeze-dried sample suspended in 30.0 mL of deionized water (DI water)) were added to a vial and mixed. The mixture was then incubated at 25 °C for 1 h and stored in the dark. The absorbance was measured at 725 nm using a UV/Vis spectrophotometer (Genesys 10S UV-Vis, Thermo Scientific, Waltham, MA, USA). The results were expressed as mg of gallic acid (GA) per gram of sample (mg GA/g sample).

#### 2.2.2. Quantification of Bixin

The previously diluted annatto seed extract (~0.1 g of freeze-dried sample suspended in 30.0 mL of DI water) was again diluted with acetone to obtain an absorbance below 1.0 at 486 nm employing a UV/Vis spectrophotometer (Genesys 10S UV-Vis, Thermo Scientific, Waltham, MA, USA). The mg of bixin per gram of sample was calculated from a calibration curve of bixin standard built between 12.5 mg/L and 0.19 mg/L [37].

#### 2.2.3. Ferric Reducing Antioxidant Power (FRAP)

The FRAP method was employed with minor modifications [38]. First, 100 μL of sample (~0.1 mg of freeze-dried sample suspended in 30.0 mL of DI water) were mixed with 900 μL of DI water and 2.0 mL of the FRAP reagent containing 10 mM solution of 2,4,6-tri-(2-pyridyl)-1,3,5-triazine (TPTZ), 20 mM solution of FeCl_3_-6H_2_O, and 0.3 mM acetate buffer at a pH 3.6 that were mixed in a 1:1:10 ratio, respectively. The mixture was then incubated at 37 °C for 30 min. The absorbance readings were taken at 593 nm using a UV/Vis spectrophotometer (Genesys 10S UV-Vis, Thermo Scientific, Waltham, MA, USA). A Trolox^®^ calibration curve was used. The results were expressed as μmol Trolox/kg.

#### 2.2.4. Superoxide Radical Scavenging Activity (SRSA)

The superoxide anion scavenging activity was measured employing the method described by Robak and Gryglewski [39]. Briefly, 3.0 mL of 16 mM Tris-HCl buffer (pH 8.0), 250 μL of 0.3 mM nitroblue tetrazolium (NBT), 250 μL of 0.936 mM nicotinamide adenine dinucleotide (NADH) solution, 500 μL of sample (~0.1 g of freeze-dried sample suspended in 30.0 mL of DI water), and 250 μL of 16 mM Tris-HCl buffer (pH 8.0) were mixed in that sequence. Subsequently, 250 μL of 0.12 mM phenazine methosulfate (PMS) solution were added to the mixture to start the reaction. The reaction continued for 5 min at 25 °C. The absorbance was taken at 560 nm employing a UV/Vis spectrophotometer (Genesys 10S UV-Vis, Thermo Scientific, Waltham, MA, USA). A Trolox^®^ calibration curve was then used. The results were expressed as μmol Trolox/kg.

#### 2.2.5. Antimicrobial Activity of Annatto Seed Extracts

The antibacterial activity against *Bacillus cereus* and *Staphylococcus aureus* was determined using the colorimetric microdilution method. First, ~0.1g of freeze-dried sample was suspended in 30.0 mL of DMSO:DI water at a 1:1 ratio. Samples were then diluted to obtain a final point well concentration from ~4.096 mg/L to ~16 mg/L, starting from 20 μL of the sample, 220 μL of nutrient broth, and 10 μL of microorganism having an absorbance between 0.1 and 0.2 in nutrient broth to 630 nm previously incubated to 37 °C for 4 h. The inhibitory concentration (IC) was taken as the concentration of the well that did not show any color change by the reaction with 3-(4,5-dimethylthiazol-2-yl)-2,5-diphenyl-tetrazolium bromide (MTT) (800 mg/L) (from yellow to purple) in the microplates [1,37]. The percentage of inhibition was determined at 630 nm using a microplate reader (ELx808, Biotek Instruments Inc., Winooski, VT, USA). The percentage of inhibition was determined using equation (Equation (1)) as follows:

(1) $$Inhibition\;\left(\%\right)=100\left(\%\right)-\frac{As\times 100}{Ac1+Ac2}$$

where *As*, *Ac*1, and *Ac*2 correspond to the sample absorbance, sample without microorganism absorbance, and microorganism in the nutritious broth absorbance, respectively.

### 2.3. Extraction of Proteins from Quinoa and Lentils

Proteins were extracted in a shaker for 12 h with 0.1 M Tris-HCl buffer at pH of 10 at 25 °C. Quinoa proteins (QP) were extracted at a buffer-to-sample ratio of 1:5 (*v*:*w*), and lentil proteins (LP) were extracted at a buffer-to-sample ratio of 1:10 (*v*:*w*). Subsequently, the samples were filtered and the solids were separated. The proteins in the supernatant were precipitated with ammonium sulfate to cause saturation without producing denaturation. Afterward, the solution was centrifuged at 12,000 *g* for 10 min (Hermle Z206A, HERMLE Labortechnik GmbH, Wehingen, Germany) followed by dialysis using a 3 kDa cellulose membrane (Fisherbrand™, Sunnyvale, CA, USA) for two days against distilled water (Type II water). DI water was changed every 12 h [40]. Isolate proteins were freeze-dried until use.

### 2.4. Preliminary Encapsulation Studies

These preliminary tests were performed to choose the encapsulation conditions that were best suited to the ionic gelation mechanism. Heating treatment at 80 °C (10–120 min), solution pH (8–11), protein solution concentration (2.5–10.0%), and calcium chloride levels (0–10%) were taken as independent variables and the empty capsule formation was the process response variable. These tests showed that the best encapsulation conditions were obtained with a 10% protein, 10% calcium chloride solution, and 60 min of heating time. SP, QP, and LP were best gelled at a pH of 9.0, whereas animal proteins (SCP) were best gelled at a pH of 11 [41].

### 2.5. Encapsulation of Annatto Extract

The SP, SCP, QP, and LP extracts (10% of protein, *w*/*v*) were suspended in DI water and stirred at room temperature for 12 h. The pH of the dispersions was adjusted to pH 9.0 with 0.2 M NaOH for SP, QP, and LP, whereas SCP was adjusted to pH 11.0. The dispersions were heated at 80 °C for 1 h. Subsequently, the annatto extract at a 1:5 core/wall ratio was added, using a homogenizer (Ultra-Turrax T18, IKA-Labortechnik, Staufen, Germany) operated at 8000 rpm for 1 min. Once the samples were homogenized, they were manually extruded using a 21-gauge needle syringe on a 10% solution of CaCl_2_. The capsules thus formed were allowed to stand in the solution for 15 min. Afterward, the capsules were immersed in a 0.01% glutaraldehyde solution for 1 min and washed with DI water, filtered (11 µm of cellulose), and freeze-dried. The capsules were characterized for encapsulation efficiency, particle size, infrared transmission spectroscopy (PerkinElmer II FT-IR spectrometer, Waltham, MA, USA), and confocal microscopy (TCS SP5 II, Leica Microsystems, Exton, PA, USA). The stability of both the encapsulated polyphenol compounds (according to Section 2.2.1.) and the bixin (according to Section 2.2.2.) were analyzed at different storage temperatures (4 °C, 23 °C, and 65 °C) for 12 d (days 0, 4, 8, and 12). The samples were tested every 4 d. Furthermore, the antimicrobial and antioxidant activities were assessed at the initial and final stages of storage, by diluting the samples in 1.0% of sodium citrate:DMSO (1:1) solution (~0.1 g of freeze-dried sample suspended in 30 mL of solution) as described previously.

#### 2.5.1. Encapsulation Efficiency

An indirect methodology determined the encapsulation efficiency of annatto extract for the bixin compound taken as the primary carotenoid from the extract [42]. The concentration of the bixin in the dispersions of the coating material and extract was determined before encapsulation (*m_a_*). Once the encapsulation process took place, these compounds were quantified again in the supernatant (*m_b_*). The encapsulation efficiency of the extract was calculated using the following equation (Equation (2)):

(2) $$\;EE=\frac{m_{a}-m_{b}}{m_{a}}\times 100\%$$

#### 2.5.2. Confocal Microscopy and Scanning Electron Microscopy (SEM)

A broadband confocal microscope (TCS SP5 II, Leica Microsystems, Exton, PA, USA) was used to acquire the confocal images of the capsules. LASAF^®^ (Leica application suite) software (4.0 version, Exton, PA, USA) was used for image analysis. The fluorescence excitation light with the 488 nm argon laser line was collected in two separate emission channels of the selected image planes, namely one from 400 to 500 nm for intrinsic auto-fluorescence and the other from 500 to 600 nm to resolve the distribution of extract within the samples [43]. SEM images were obtained on the lyophilized capsules, which were fixed with an adhesive layer of graphite and coated with gold. The microphotographs were taken using thermionic equipment (JEOL-JSM 6490LV, Jeol, Peabody, MA, USA) at room temperature and with a 20 kV acceleration voltage [44].

#### 2.5.3. Particle Size Measurements

The particle size was calculated by digital image analysis [44]. Photographs of the microcapsules were taken with a digital camera (Huawei, Chaoyang, District, Beijing, China), and approximately 1000 particles were analyzed per image to determine the size using ImageJ^®^ software (1.52a version, Wayne Rasband, Madison, WI, USA) [44].

#### 2.5.4. Infrared Transmission Spectroscopy

The dry sample of annatto seed extract, the coating materials, and the encapsulated extracts were characterized by Fourier transform infrared spectroscopy (FT-IR). A PerkinElmer II FT-IR spectrometer (Waltham, MA, USA) was employed. The infrared spectra were taken in a range from 4000 to 400 cm^−1^ (0.5 cm^−1^ of resolution) in duplicate for each sample.

### 2.6. Statistical Analysis

The results are presented as means of three replicates and standard deviation (SD), according to the normality of the data. The analysis was performed using Statgraphics^®^ Centurion XVI software (XVI version, StatPoint Technologies, Inc., Warrenton, VA, USA). Differences among treatment means were tested using Fisher’s least significant difference (LSD) test (*p* < 0.05).

## 3. Results

### 3.1. Encapsulation of Annatto Extract using Plant Proteins as a Wall Material

**Figure 1:**
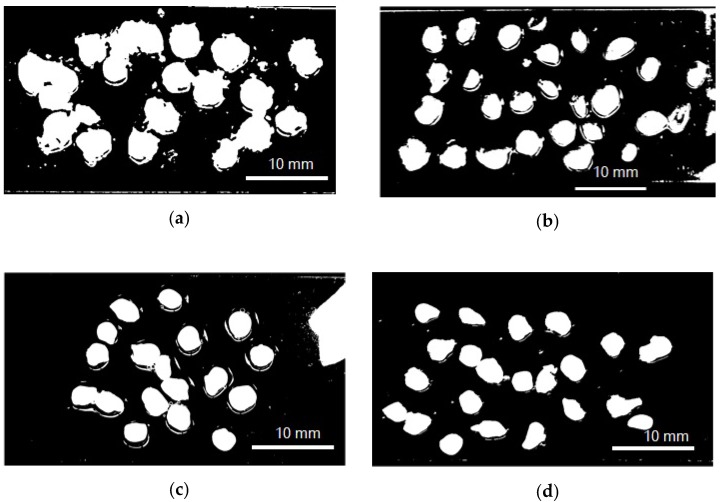
Model photograph of encapsulated annatto extract in solution used to determine particle size. (**a**) Encapsulated extract with lentil proteins, (**b**) encapsulated extract with quinoa proteins, (**c**) encapsulated extract with sodium caseinate, and (**d**) encapsulated extract with soy proteins.

The encapsulation efficiency and particle size (Figure 1) (wet and dry basis) of the encapsulated extract are listed in Table 1. Encapsulation efficiencies had statistically significant differences (*p* < 0.05) when different protein sources were employed. The most significant values of the encapsulation efficiency were achieved by SCP, followed by SP, LP, and QP. There were no statistically significant differences in particle size neither between capsules in solution and freeze-dried capsules, nor different carrier proteins used.

**Figure 2:**
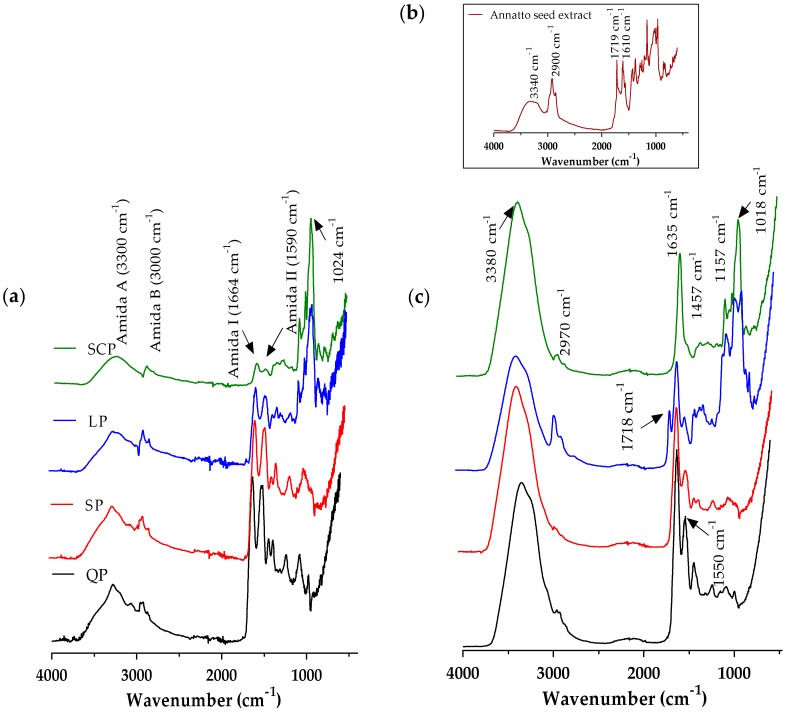
Fourier transform infrared spectroscopy (FT-IR) spectrum average of (**a**) proteins, (**b**) free annatto extract, and (**c**)the encapsulated annatto extract with the different carrier materials. LP: lentil protein, QP: quinoa protein, SCP: sodium caseinate, SP: soy protein.

Once the annatto extract capsules were obtained by the ionic gelation method, the FT-IR spectra of the proteins, the annatto extract, and the encapsulated extract with the different carrier materials were evaluated (Figure 2). These spectra confirm the presence of annatto seed extract within the capsule.

**Figure 3:**
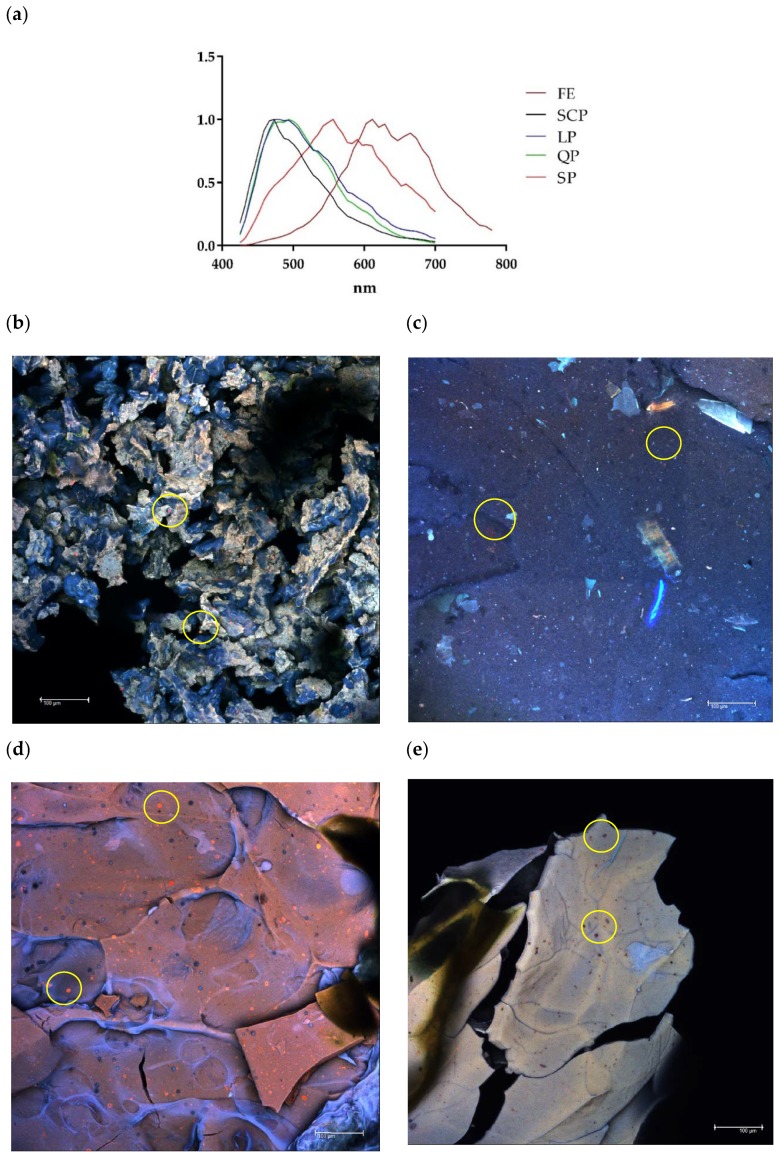
Confocal laser scanning microscopy (CLSM). (**a**) Fluorescence spectrum of free and encapsulated annatto extract; (**b**) encapsulated extract with lentil proteins, (**c**) encapsulated extract with quinoa proteins, (**d**) encapsulated extract with sodium caseinate, and (**e**) encapsulated extract with soy proteins. FE: free extract, LP: lentil protein, QP: quinoa protein, SCP: sodium caseinate, SP: soy protein, scale bar: 100 μm.

Similarly, the presence of the protected extract within each encapsulated material was confirmed by the confocal microscopy images (Figure 3). Based on the confocal fluorescence images (Figure 3), the particles in the annatto seed extract are widely distributed and embedded within the wall structure and seen as the small red particles. The annatto seed extract shows a peak with maximum intensity between 600 and 700 nm, while the self-fluorescence in larger areas was identified as protein, emitting a blue-green coloration with a maximum intensity between 450 and 500 nm in the case LP and QP, and between 500 and 600 nm for SP (Figure 3a).

**Figure 4:**
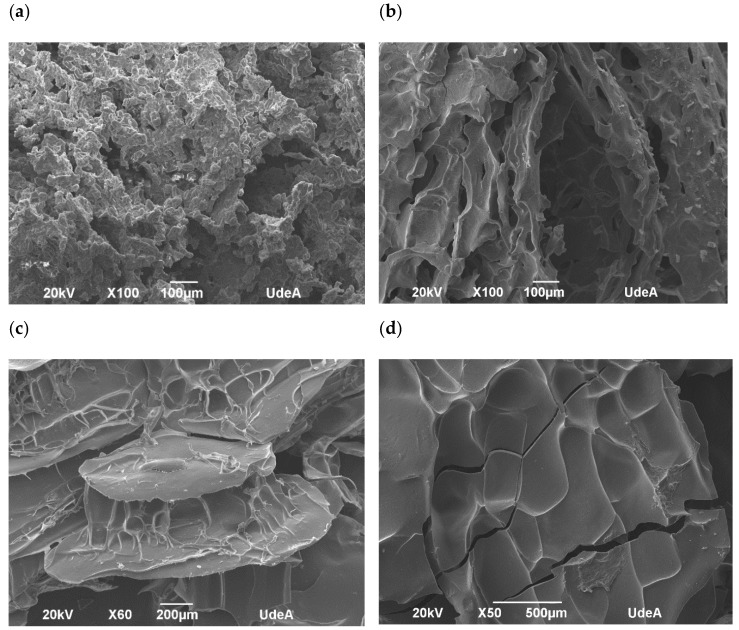
Scanning electron microphotographs of encapsulated annatto extract. (**a**) Encapsulated extract with lentil proteins, (**b**) encapsulated extract with quinoa proteins, (**c**) encapsulated extract with sodium caseinate, and (**d**) encapsulated extract with soy proteins.

The morphological analysis of the encapsulated annatto seed extract is depicted in the SEM images. Major differences can be observed on the surface of the obtained capsules (Figure 3). In fact, the lentil proteins (Figure 4a) exhibited the largest morphological differences between the samples studied.

### 3.2. Stability Studies of the Encapsulated Annatto Seed Extract

**Figure 5:**
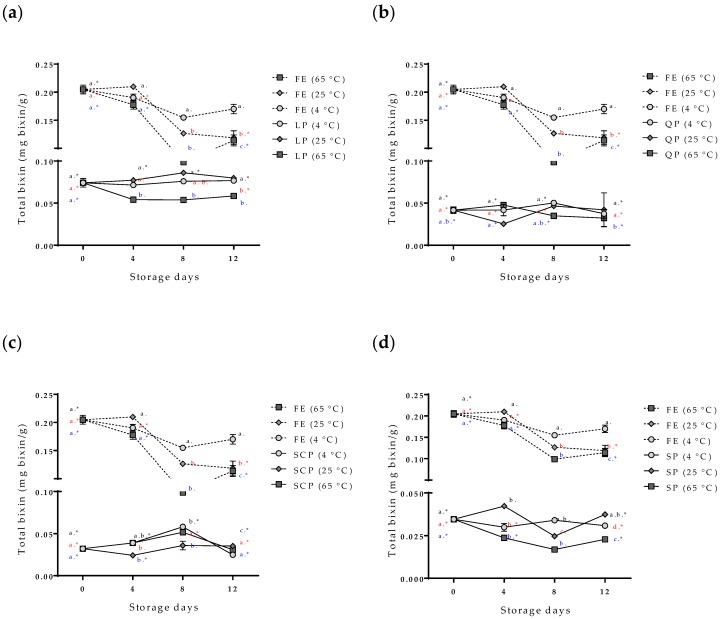
Effect of storage time and temperature of bixin isolated from the free and encapsulated annatto extracts. (**a**) Free and encapsulated extracts with lentil proteins, (**b**) free and encapsulated extracts with quinoa proteins, (**c**) free and encapsulated extracts with sodium caseinate, and (**d**) free and encapsulated extracts with soy proteins. FE: free extract, LP: lentil protein, QP: quinoa protein, SCP: sodium caseinate, SP: soy protein. Different superscript letters (a–c) within figure indicate significant differences between storage days in the same sample (*p* < 0.05) and samples marked with “∗” are not significantly different between the samples at same storage day (*p* < 0.05). Both differences according to Fisher’s least significant difference (LSD-Fisher) test.

**Figure 6:**
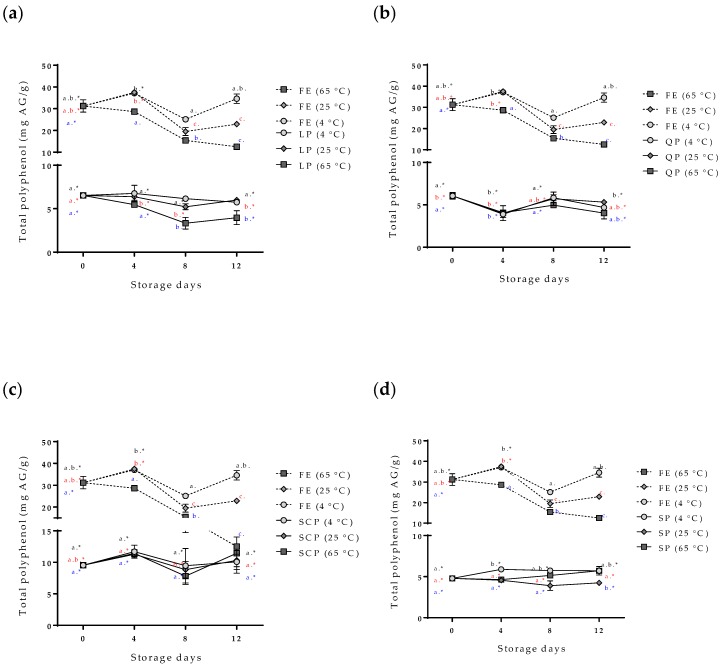
Effect of storage time and temperature of total polyphenol compounds isolated from the free and encapsulated annatto extracts. (**a**) Free and encapsulated extracts with lentil proteins, (**b**) free and encapsulated extracts with quinoa proteins, (**c**) free and encapsulated extracts with sodium caseinate, and (**d**) free and encapsulated extracts with soy proteins. FE: free extract, LP: lentil protein, QP: quinoa protein, SCP: sodium caseinate, SP: soy protein. Different superscript letters (a–c) within figure indicate significant differences between storage days in the same sample (*p* < 0.05) and samples marked with “∗” are not significantly different between the samples at same storage day (*p* < 0.05). Both differences according to Fisher’s least significant difference (LSD-Fisher) test.

The evaluation of the stability of the bioactive compounds (the bixin and the total polyphenol compounds) of the free and encapsulated annatto seed extracts is shown in Figure 5 and Figure 6. The content of the total polyphenol compounds and the bixin in the free extract showed a slight decrease in storage time at 4 °C, but it had no statistically significant differences (*p* > 0.05). On the other hand, at temperatures of 25 °C and 65 °C, the content of bioactive compounds decreased during storage, and 65 °C was the temperature with the greater effect. The bixin went from 0.205 ± 0.008 to 0.114 ± 0.010 mg bixin/g during the 12 days, and from 31.25 ± 7.83 mg AG/g of samples to 12.52 ± 0.34 mg AG/g of samples for the polyphenolic compounds during the same storage time. In the case of the extract encapsulations, bioactive compounds showed a lower concentration within the capsules but remained stable independent of the storage temperature, SCP being the most stable followed by SP, QP, and LP.

### 3.3. Antioxidant and Antimicrobial Activities of the Encapsulated Annatto Extract

Table 2 and Figure 6 show the antioxidant and antimicrobial activities of the annatto seed extract free and encapsulated for 0 d and 12 d stored to 4, 25, and 65 °C. The stability of these biological activities (Figure 5 and Figure 6) showed similar behavior to the stability of the phenol compounds and the bixin reported in Figure 4 and Figure 5. Both biological activities were closely related to bioactive compounds present in the annatto extract [37].

The antioxidant activity conducted by the FRAP and SRSA methods of encapsulated extracts showed a more stable behavior than those obtained for the free extract, irrespective of the carrier material used. However, the antioxidant power of the encapsulated extracts was less than the free extract.

**Figure 7:**
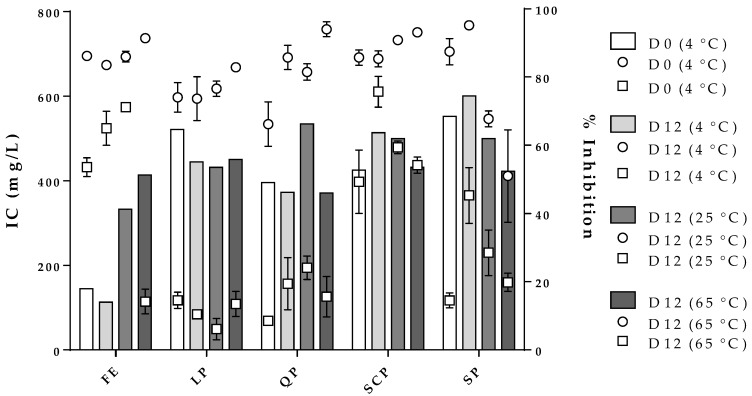
Effect of storage conditions on the free and encapsulated extracts and their influence on the inhibitory concentration (IC, bars, y-axis) against *B. cereus* (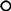, z-axis) and *S. aureus* (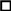, z-axis). FE: free extract, LP: lentil protein, QP: quinoa protein, SCP: sodium caseinate, SP: soy protein, D0: zero-day storage, D12: twelve-day storage.

Figure 7 shows the effect of storage time and temperature on the inhibition concentration of the free and encapsulated extracts for the two strains evaluated at a constant percentage of inhibition (% inhibition). In the case of the free extract, an IC of 180 mg/L (bars, y-axis) was calculated to obtain an inhibition percentage of 60% for *S. aureus* and 90% for *B. cereus* (z-axis) for zero-day storage. After the twelve storage days at the three temperatures studied, the free extract stored at 4 °C reported an IC and a percentage of inhibition of *S. aureus* and *B. cereus* comparable with zero-day storage. On the other hand, the free extracts stored at 25 °C and 65 °C needed to increase significantly their concentration in order to continue to obtain inhibition percentages similar to zero-day. The free extract stored at 65 °C could not keep the inhibition for *S. aureus* constant even though its concentration was increased, a situation that did not occur for *B. cereus.* In the case of the encapsulated extract, the IC for both microorganisms exhibited a consistent behavior independent of the protein type used as a carrier material. Moreover, the results obtained showed the inhibition percentages were always higher for *B. cereus* than for *S. aureus* for all treatments.

## 4. Discussion

The results indicated that the encapsulation of the annatto extract by ionic gelation using LP, QP, SP, and SCP as carrier materials affected the performed assays. For the encapsulation process, the protein dispersions use their moieties of phosphoserine, COO– (glutamic acid and aspartic acid), and thiol groups (cysteine) to react with the calcium ions present in the gelling solution used in the process encapsulation by ionic gelation. These reactions form a hydrogel and encapsulate the bioactive compounds of the annatto extract simultaneously [30,45]. Comparing the sum of the percentages of the phosphoserine and amino acid groups that favor the formation of hydrogels for each protein used, we found that SP (~36.0%) was the protein with the largest amino acid content that promotes the formation of hydrogels, followed by SCP (~32.2%), LP (~29.3%), and QP (~26.0%) [46,47,48,49]. Consequently, the material with the highest content of reactive groups for crosslinking the hydrogel forms a hydrogel at a higher reaction rate, with greater structure and encapsulating the extract more efficiently [30]. This explains the higher percentage of SP encapsulation compared to QP and LP. However, by comparing SP and SCP, even though SCP contains a lower percentage of relevant amino acids in the ion gelation–encapsulation process, SCP had a greater encapsulation efficiency than SP. The difference between the secondary structures of the proteins used is a key factor in the encapsulation process. According to reported studies, either SP or QP and LP are globular proteins, where their secondary structure is governed by β-sheet and α-helix, and where many of their amino acids are hidden inside the structure. Therefore, the denaturation process of proteins is essential to exposing the amino acids for their reaction with Ca^2+^ and, as a consequence, to inducing the hydrogel formation process [30,39,50]. On the other hand, the micellar structure of SCP allows the radical groups of their amino acids to be more exposed and to react with the medium [46].

FT-IR spectra were obtained for the carrier materials (Figure 2a). However, the observed broadband of emissions between 3000 and 3500 cm^−1^ corresponds to the O–H and N–H groups exposed and free to react with intermolecular and intramolecular hydrogen bonds and some COO– groups of amino acids in the protein structure. The characteristic peaks corresponding to the sum of amide I (1664 cm^−1^) and amide II (1590 cm^−1^) are related to the secondary structure of each protein source and the C–N stretching and N–H flat flexion, respectively [24]. It was observed that proteins from plant sources have amide I and II bands with greater intensity than those of SCP, according to the analysis of the secondary structure previously raised, and that the plant proteins had more folds such as β-sheet, α-helix, and random coils [51]. The band at 1024cm^−1^ is formed by the C-H bending outside the plane (of aromatic structures) and the PO^−2^ or P–OH stretch of the phosphate esters, which are present in serine residues of proteins [24]. The infrared spectrum of the annatto seed extract (Figure 2b) showed absorption peaks at 3340 cm^−1^, 2900 cm^−1^, 1719 cm^−1^, and 1610 cm^−1^ corresponding to OH groups, H–C–H links, and C–O moieties present in the bixin and polyphenol compounds of the OH extract of annatto seeds [52]. The infrared spectrum of the encapsulated annatto extract (Figure 2c) demonstrated the synergy between the infrared spectra obtained for each encapsulation material and the one obtained for the free extract, demonstrating that the powder contained the bioactive compounds of the extract and the encapsulation material.

Results from confocal and SEM photographs show the extract of annatto seed encapsulated with the different carrier material used. Figure 3b shows the annatto encapsulated with LP, which consists of a structure of small irregular agglomerated hydrogels, where the annatto extract is heterogeneously distributed throughout the structure. On the other hand, Figure 3c–e shows denser and more homogeneous gel structures, which correspond to the encapsulated extract with QP, SCP, and SP, respectively. These results are consistent with those reported in previous literature, where globular proteins are shown forming gels with irregular surfaces and consist of protein aggregates [53]. However, quinoa and soy proteins can form gels with more uniform polymerization with a flat, regular surface [53,54,55]. These morphological characteristics obtained are confirmed in the scanning electron microscopy images presented in Figure 4.

The variability and increase in the content of total polyphenols in the capsules during the storage time (Figure 6) are possible due to the hydrolysis of the conjugated polyphenols of annatto seed extract during the degradation; free hydroxyl groups are released, resulting in an increased value obtained for the Folin–Ciocalteu method, sensitive to free hydroxyl groups [24,25]. This same behavior has been reported for other extracts rich in polyphenol compounds that have been encapsulated with proteins as carrier materials [24,25,26]. Moreno et al. encapsulated grape marc phenolics using the spray-drying technique, evaluating the use of maltodextrin, whey protein isolate, and pea protein isolate as carrier agents and their effect on process performance and thermal stability of phenolic compounds [26]. They reported that the pea protein microcapsules showed the lowest degradation rate for the bioactive compounds (longest half-life periods) compared to the other two carrier agents at temperatures of 40 °C (11-month half-life period) and 60 °C (9-month half-life period) of storage. They concluded that proteins are interesting alternatives to maltodextrin frequently used in microencapsulation processes, highlighting that although the use of pea proteins as carrier agents does not offer high yields, it offers excellent thermal stability of polyphenolic compounds, coinciding with the results obtained in this study [26]. The bixin was more stable (*p* < 0.05) for SP- and SCP-encapsulated extracts than for LP and QP encapsulations. However, the bixin content at zero-day storage had differences between the carrier agents (Figure 6). The extract encapsulated with SCP as carrier material reported a lower value of bixin content (0.032 mg bixin/g) than extracts encapsulated with the other materials such as LP (0.074 mg bixin/g), despite having been formulated with the same amount of extract. This behavior could possibly be attributed to the complex formations between the extract and the carrier materials by physical forces such as hydrophobic, electrostatic, Van der Waals, and hydrogen-bonding forces, being more or stronger with SCP than with the other proteins used, thereby affecting the extraction of bixin in the quantification process [23].

The percentage of bixin degradation for the LP-encapsulated extract was ~39.17%, the highest among the encapsulated extracts. This is possibly due to the structure of the capsule where, unlike the other coating materials used, it had a rougher and more heterogeneous structure than the other capsules (Figure 4a) formed by small agglomerates. This structure allows for a greater area of exposure to external conditions, decreasing the effectiveness of the protection of the bioactive compound.

Regarding the stability of the antioxidant (Table 2) and antimicrobial (Figure 7) activities of the free and encapsulated extracts, for both bioactivities the free extract presented figuratively significant differences in storage conditions studied, especially at high temperatures. On the other hand, the encapsulated extract, indifferent to the coating material used, had higher stability for antioxidant and antimicrobial activities, which were closely related to the stability of the bioactive compounds of the free extract (Figure 5 and Figure 6). The stability results of antimicrobial and antioxidant activities of the encapsulated extract are comparable to those reported by some authors [24,25].

## 5. Conclusions

This study developed the use of lentil and quinoa proteins in microencapsulation of annatto seed extract using the ionic gelation method. The results show that different plant proteins have a significant effect (*p* < 0.05) on the microencapsulating efficiency of annatto extracts. With respect to the protection of the annatto seed extract at different storage temperatures, proteins managed to protect and give stability to the antioxidant and antimicrobial activities of the extract, as well as to the bioactive compounds to which they attributed such bioactivities, even up to a storage temperature of 65 °C for 12 d. These results are essential for the use of plant proteins as coating materials in asset encapsulation processes. However, more studies are needed to improve the encapsulation efficiencies and the particle sizes of the encapsulated products with plant proteins, adding potential to their use in the food or pharmaceutical industries.

## Figures and Tables

**Table 1:** Comparison of the encapsulation efficiency and particle size of encapsulated annatto extracts.

Sample		LP	QP	SCP	SP
EE (Bixin)	(%)	68.61 ± 2.38 ^a^	58.38 ± 5.55 ^b^	79.36 ± 0.82 ^c^	71.88 ± 0.97 ^a^
Particle size, in solution	mm	4.00 ± 2.90 ^a,^*	4.08 ± 3.03 ^a^	3.67 ± 0.41 ^a^	3.52 ± 2.60 ^a^
Particle, freeze-dried	mm	2.08 ± 1.50 ^a,^*	2.00 ± 0.96 ^a^	2.61 ± 0.94 ^a^	2.84 ± 0.79 ^a^

Different superscript letters (a–c) within rows indicate significant differences between samples (*p* < 0.05) according to Fisher’s least significant difference (LSD-Fisher) test; values are expressed as mean ± standard deviation (SD) (*n* = 3). LP: lentil protein, QP: quinoa protein, SCP: sodium caseinate, SP: soy protein, EE: encapsulation efficiency. Samples marked with “∗” are not significantly different at *p <* 0.05 between treatment.

**Table 2:** Comparison of the antioxidant activity of free and encapsulated annatto extracts stored under different conditions.

Antioxidant Activities (µmol Trolox/Kg)	Sample				
	FE	LP	QP	SCP	SP
FRAP D0	157.32 ± 3.61 ^a^	8.05 ± 0.30 ^a^	8.00 ± 0.20 ^a^	10.49 ± 0.43 ^a^	12.56 ± 0.37 ^a^
FRAP D12 (4 °C)	128.08 ± 14.07 ^b^	9.74 ± 1.11 ^a^	8.82 ± 3.94 ^a^	8.19 ± 0.24 ^a^	10.07 ± 2.12 ^a^
FRAP D12 (25 °C)	102.83 ± 11.57 ^b^	10.33 ± 0.47 ^a^	12.02 ± 3.12 ^a^	10.69 ± 2.56 ^a^	10.14 ± 1.81^a^
FRAP D12 (65 °C)	73.52 ± 10.44 ^c^	5.64 ± 0.62 ^b^	8.67 ± 2.44 ^a^	9.94 ± 3.09 ^a^	12.08 ± 1.77 ^a^
SRSA D0	13.27 ± 2.54 ^a^	2.78 ± 0.16 ^a^	3.77 ± 0.34 ^a^	3.87 ± 0.35 ^a^	3.29 ± 0.09 ^a^
SRSA D12 (4 °C)	12.38 ± 0.12 ^a^	2.80 ± 0.13 ^a,b^	2.58 ± 0.11 ^a,b^	3.42 ± 0.61 ^a^	3.98 ± 0.13 ^a^
SRSA D12 (25 °C)	8.24 ± 0.65 ^b^	4.12 ± 0.30 ^b,c^	4.38 ± 0.84 ^b,c^	4.02 ± 0.80 ^a^	3.37 ± 0.22 ^a^
SRSA D12 (65 °C)	5.94 ± 0.46 ^c^	3.17 ± 0.03 ^c^	2.39 ± 0.02 ^c^	3.85 ± 0.30 ^a^	4.52 ± 1.04 ^a^

Different superscript letters (a–c) within columns and tests indicate significant differences between storage conditions (*p* < 0.05) according to the LSD-Fisher test; values are expressed as mean ± standard deviation (*n* = 3). FE: free extract, LP: lentil protein, QP: quinoa protein, SCP: sodium caseinate, SP: soy protein, FRAP: ferric reducing antioxidant power, SRSA: superoxide radical scavenging activity, EE: encapsulation efficiency, D0: zero-day storage, D12: twelve-day storage.
